# Conditional disease-free survival after liver transplantation for hepatocellular carcinoma: A two-center experience

**DOI:** 10.1097/MD.0000000000004383

**Published:** 2016-08-07

**Authors:** Jian Dong, Ying Zhu, Feng Ma, Yifang Ren, Jianwen Lu, Zhengxin Wang, Lunxiu Qin, Rongqian Wu, Yi Lv

**Affiliations:** a Department of Hepatobiliary Surgery, First Affiliated Hospital of Medical College, Xi’an Jiaotong University; b Institute of Advanced Surgical Technology and Engineering, First Affiliated Hospital of Medical College, Xi’an Jiaotong University, Xi’an, Shaanxi Province; c Department of Surgery, Huashan Hospital; d Cancer Metastasis Institute, Fudan University, Shanghai, P.R. China.

**Keywords:** conditional disease-free survival, hepatocellular carcinoma, liver transplantation

## Abstract

Traditionally, survival estimates following liver transplantation (LT) of hepatocellular carcinoma (HCC) patients were calculated as survival from the surgery date, but future survival probabilities can change over time and conditional disease-free survival (CDFS) may provide patients and clinicians with more accurate prognostic information. This study aimed to assess CDFS in HCC patients after LT.

Three hundred eighty-four HCC patients who underwent LT were included. Disease-free survival (DFS) was calculated using the Kaplan–Meier analysis. The 3-year CDFS, which represents the probability of remaining disease free for an additional 3 years, was calculated.

1-, 3-, and 5-year DFS rates after LT were 69.9%, 45.8%, and 39.0 %, respectively. Based on the concept of CDFS, the probability of surviving an additional 3 years given that the patient was disease free at 1 year, 3 years, and 5 years were 58.4%, 76.9%, and 83.1%, respectively. Multivariate analysis indicated that larger tumor size (hazard ratio [HR], 1.509; 95% CI, 1.146–1.985; *P* = 0.003) was associated with poorer DFS. Patients with worse prognostic features at baseline demonstrated the greater increase in CDFS over time.

Survival estimates following liver transplantation of HCC patients change according to survival time accrued since surgery. CDFS estimates improved dramatically over time especially among patients with worse prognostic features at the time of surgery. CDFS may be a useful tool in counseling patients with HCC, as it is a more accurate assessment of future survival for those patients who have already survived a certain amount of time.

## 1 Introduction

Hepatocellular carcinoma (HCC) is the fifth most common cancer and the third leading cause of cancer-related death.^[1,2]^ Liver transplantation (LT) has been considered to be a standard therapy for HCC patients and end-stage liver diseases.^[3]^

Generally, survival estimates are reported from the diagnosis time. These survival estimates are typically stratified using many different clinicopathologic risk factors such as vascular invasion, lymph node metastasis, tumor size, and American Joint Committee on Cancer (AJCC) staging system.^[4]^ These estimates provide patients and clinicians with important information. However, due to the fact that the risk of recurrence and death often is highest during the initial few years after surgery,^[5,6]^ this kind of survival curves may not provide a real-time prediction for survival. Conditional survival, which takes into account changes in risk over time, can offer more accurate estimates for these patients.^[7,8]^ There are many reports about conditional survival after resection for various cancers including HCC. However, there has been no report about a conditional survival after LT for HCC patients. The aim of this study, therefore, was to estimate conditional disease-free survival (CDFS) of HCC patients who underwent LT. In addition, we sought to investigate the influence of various clinicopathologic prognostic factors on disease-free survival (DFS) and CDFS among HCC patients who underwent LT.

## 2 Methods

### 2.1 Patient population and data collection

Three hundred eighty-four patients treated with LT for HCC were identified from the First Affiliated Hospital of Medical College, Xi’an Jiaotong University and Department of Surgery, Huashan Hospital, Fudan University between January 1, 2003, and December 31, 2014. The study was approved by the Institutional Review Boards of the respective institutions. HCC were confirmed by histopathologic examinations of the explanted liver on all the included patients. Standard demographic and clinicopathologic data were collected, including the following: age, sex, tumor site, tumor size. Date of last follow-up and vital status were collected on all patients.

### 2.2 Patient selection and follow-up

We selected HCC patients for LT according to the Hang Zhou criteria.^[9,10]^ However, for the alpha fetoprotein (AFP), we chose 1000 ng/mL as the cutoff level. This is based on a recent study that showed a pretransplant AFP level >1000 ng/mL predicts posttransplant HCC recurrence and applying an AFP cutoff level of 1000 ng/mL would result in a 20% reduction in HCC recurrence after transplantation.^[11]^ Before the operation, patients were assessed with a baseline history, physical examination, serum laboratory tests, and image examinations. After surgery, all patients were observed periodically at follow-up to monitor possible recurrence of HCCs. Ultrasound, serum AFP level measurement, and biochemical liver function tests were conducted 3 or 6 months after discharge. Recurrence was diagnosed on the basis of 2 coinciding imaging techniques or the combination of increased AFP levels and consistent ultrasound, computed tomographic, or MRI findings.

### 2.3 Definition of conditional survival

CDFS3 estimates were calculated as the probability of survival for an additional 3 years, given that the patient disease free at x years after surgery, calculated as: CDFS3 = DFS(x+3)/DFS(x).

Changes in CDFS3 over time were assessed using linear regression, and standardized differences (d) were used to assess the differences of CDFS3 between subgroups.^[12]^ The effect size is a measure which is independent of the sample size and can give a more robust estimation of a difference in means or proportions. d values less than 0.1 indicate very small differences between groups, d values between 0.1 and 0.3 indicate small differences, d values between 0.3 and 0.5 indicate moderate differences, and d values greater than 0.5 indicate considerable differences.^[13,14]^

### 2.4 Study variables

Baseline variables collected at inclusion for their association with demographic data: age, gender; liver function: Child-Turcotte-Pugh (CTP) score,^[15]^ model for end-stage liver disease (MELD) score;^[16]^ clinical pathologic parameters: AFP, tumor number, tumor size, capsule, microvascular invasion, differentiation, AJCC stage.

### 2.5 Statistical methods

Survival rates were calculated using the Kaplan–Meier method and the differences in survival between groups were compared using the log-rank test. The association of relevant clinicopathologic variables with DFS was assessed using Cox proportional hazards models, the variable was excluded in the univariable and multivariable Cox proportional hazards analysis. We also computed 3-year CDFS estimates within strata defined by variables that were significantly related to patient survival at the log-rank test.

All statistical analyses were carried out using the SPSS 20.0 software (SPSS Inc., Chicago, IL). All tests were 2-sided, and *P* < 0.05 was considered statistically significant.

## 3 Results

### 3.1 Demographic and clinicopathologic characteristics

Baseline characteristics of the included patients are presented in Table 1. The patients consisted of 331 males and 53 females. Their average (SD) age was 51.3 (11.0) years. Forty-two patients (10.9%) were infected with hepatitis C virus. The median tumor size was 5 (range: 1–15) cm. Most patients had solitary tumors (n = 318, 82.8 %). Complete capsule was present in 197 (51.3%) patients, and microvascular invasion occurred in 151 (39.3%) patients. One hundred forty-two patients (37.0%) met the Milan criteria.

**Table 1:**
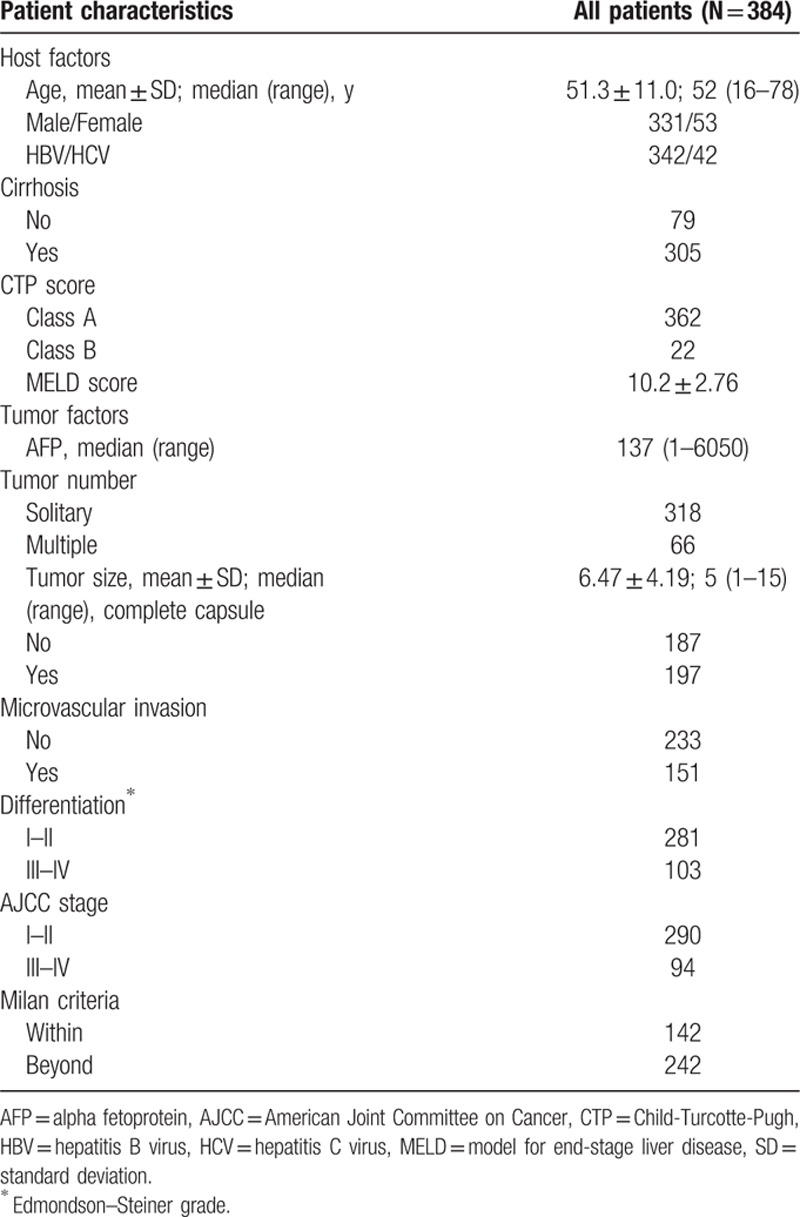
Baseline characteristics of the study population.

### 3.2 Factors associated with disease-free survival

**Figure 1:**
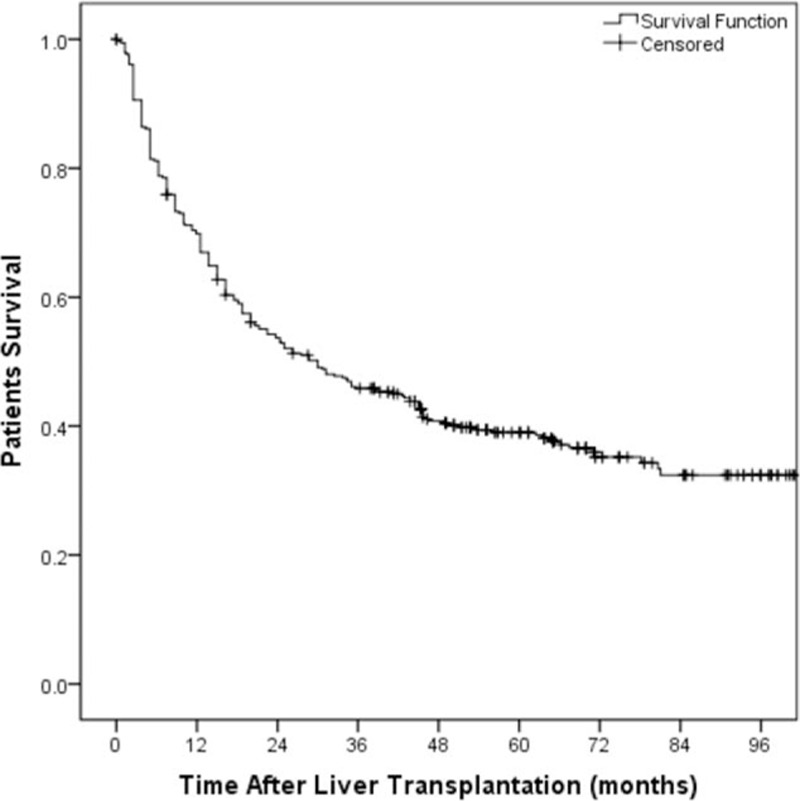
Actuarial patient disease-free survival curve of the whole study population of 384 patients submitted to liver transplantation.

Patient follow-up as of January 2015 or to the time of death ranged from 24 to 96 months. The median follow-up period was 26.3 months (range, 0–104.7 months). Two hundred thirty-seven (47.40%) patients recurred, 29 patients had late recurrence (≥3 years). Their 1-, 3-, and 5-year DFS rates were 69.9%, 45.8%, and 39.0%, respectively (Fig. 1). On the univariate analysis, the following factors were associated with unfavorable DFS: serum AFP levels (hazard ratio [HR], 1.959; 95% CI, 1.439–2.666; *P* < 0.001), tumor number (HR, 1.745; 95% CI, 1.157–2.632; *P* < 0.001), tumor size (HR, 3.303; 95% CI, 2.443–4.466; *P* < 0.001), capsule (HR, 1.598; 95% CI, 1.192–2.144; *P* = 0.002), microvascular invasion (MVI) (HR, 2.583; 95% CI, 1.877–3.552; *P* < 0.001), differentiation (HR, 1.594; 95% CI, 1.129–2.249; *P* = 0.002), AJCC stage (HR, 3.957; 95% CI, 2.599–6.024; *P* < 0.001), and Milan criteria (HR, 2.420; 95% CI, 1.799–3.255; *P* < 0.001) (Table 2). Multivariate analysis on these parameters revealed that tumor size (HR, 1.509; 95% CI, 1.146–1.985; *P* = 0.003) was independently associated with DFS (Table 3).

**Table 2:**
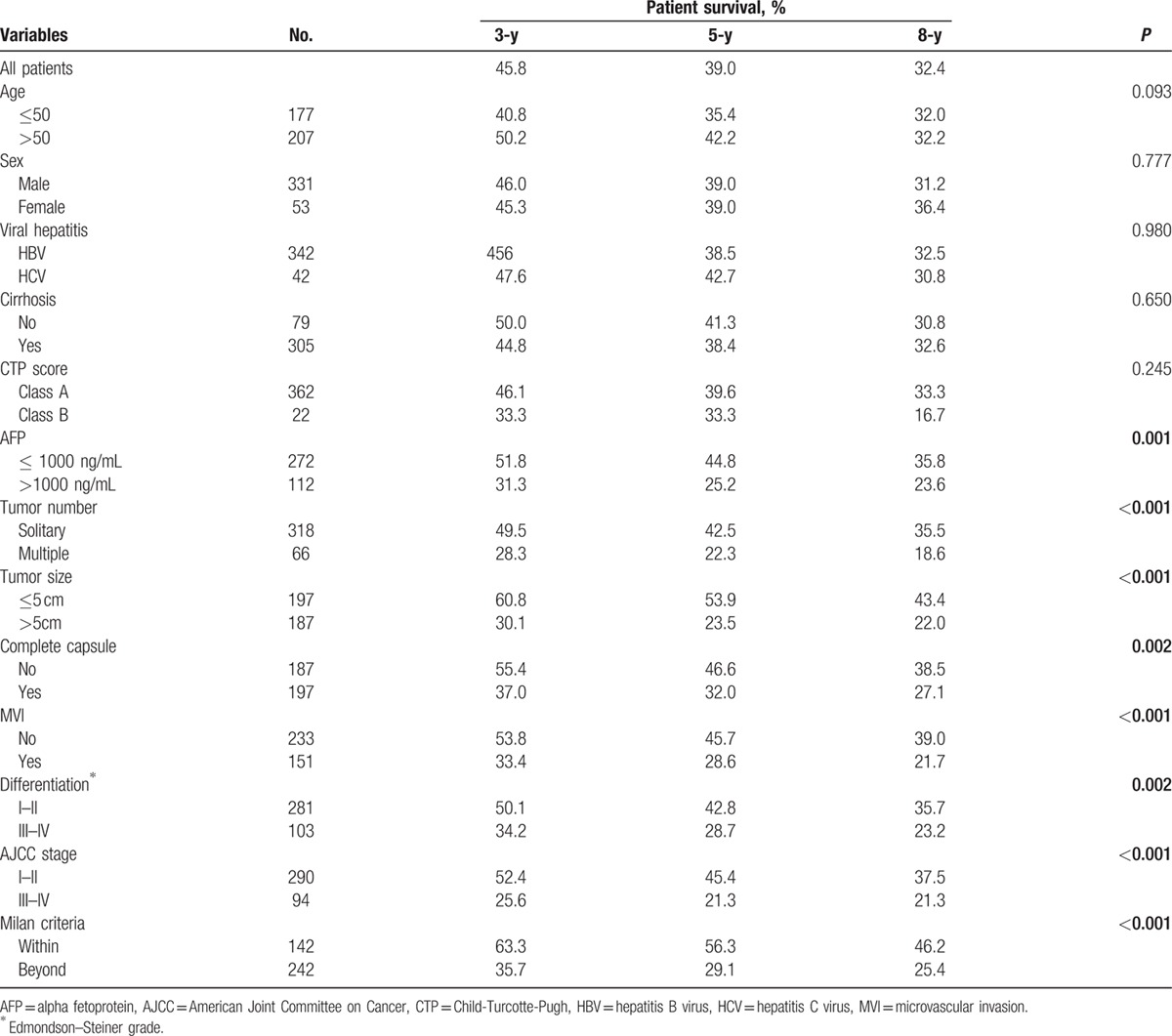
Three-year, 5-year, and 8-year disease-free survival rates in relationship to patients’ characteristics.

**Table 3:**
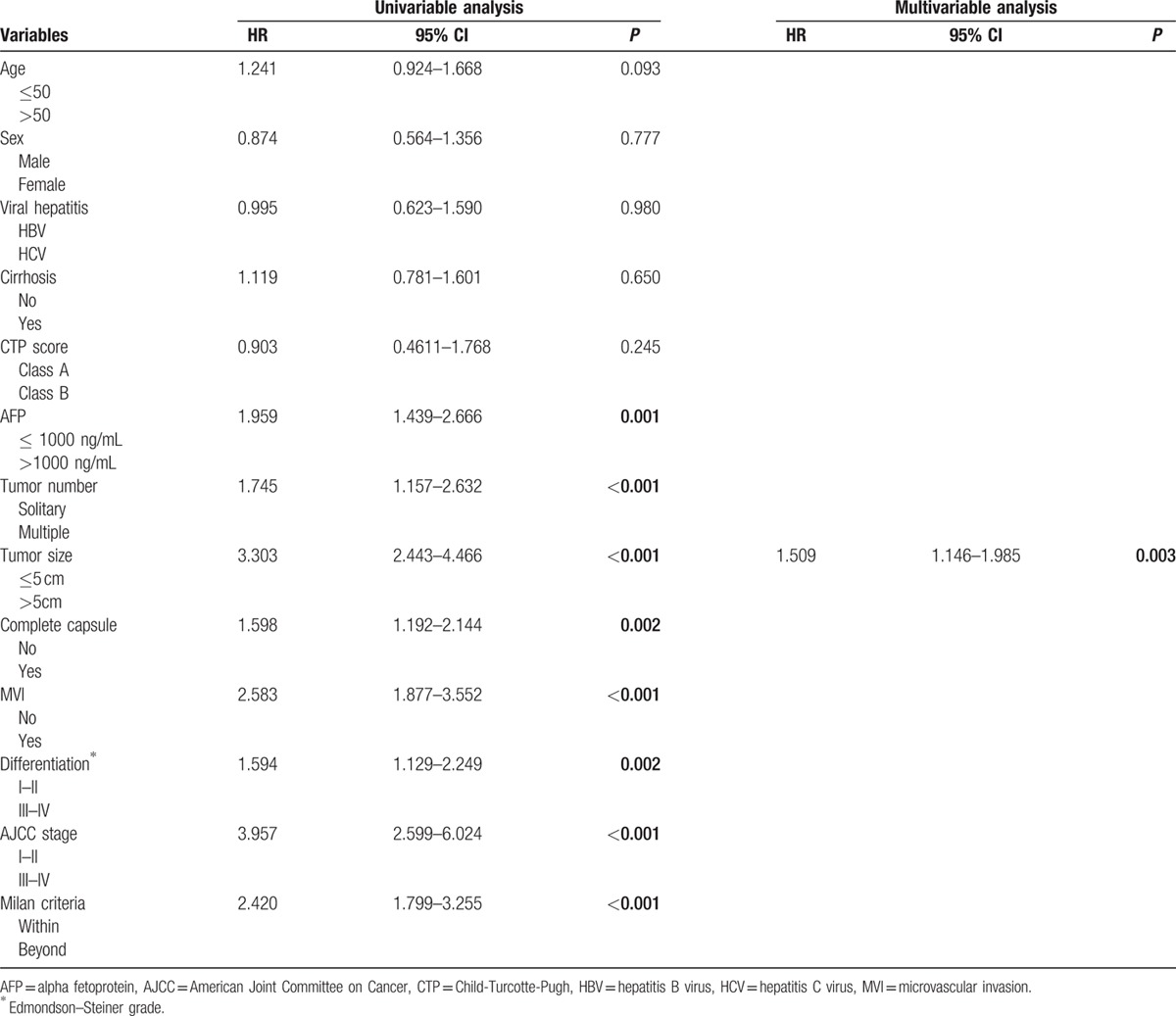
Univariable and multivariable Cox proportional hazards analysis for disease-free survival.

### 3.3 Comparison of disease-free survival and conditional disease-free survival

CDFS are summarized in Table 4. The probability of surviving an additional 3 years, given that the patient had survived for 1, 3, and 5 years was 58.4%, 76.9%, and 83.1%, respectively.

**Table 4:**
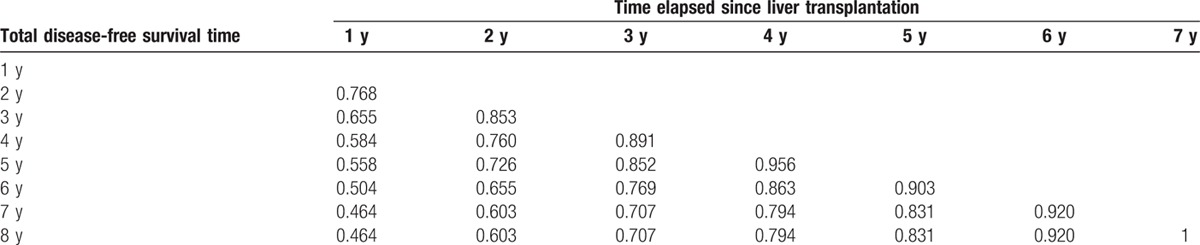
Proportion of patients who reach a certain disease-free survival time point given that they have already survived a certain amount of time.

**Figure 2:**
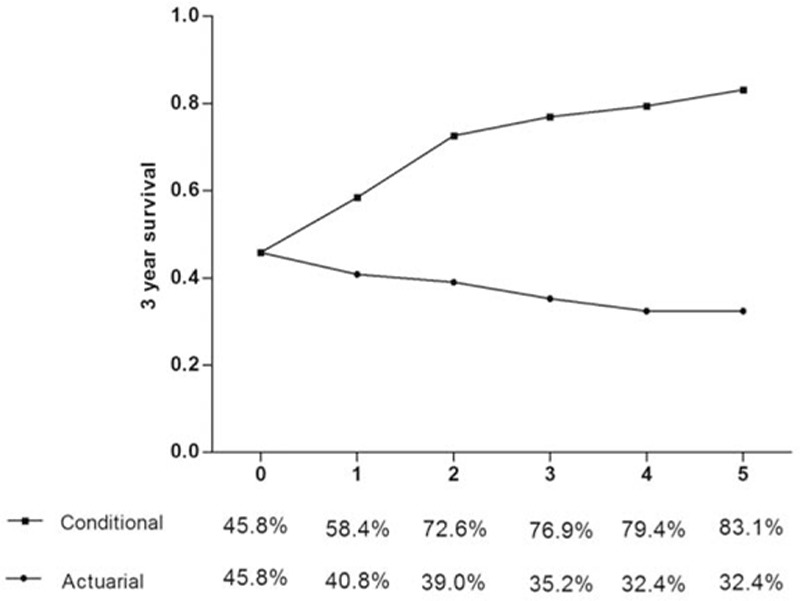
Three-year conditional disease-free survival relative to actuarial disease free-survival.

The 3-year DFS rate was 45.8 % and decreased to 35.2 % at 6 years. The 3-year CDFS at 3 years (CDFS3), the probability of surviving to postoperative year 6 without recurrence after having already survived 3 years, was 76.9 %. Similarly, the 5-year CDFS3, the probability of surviving to postoperative year 8 after having already survived 5 years, was 83.1 % as compared with an actuarial DFS 8-year rate of 32.4 %. Five-year CDFS3 rates increased over time from 45.8 % to 83.1% (*P* < 0.05), whereas actuarial DFS decreased over time from 45.8 % at 3 years to 32.4 % at 8 years (*P* < 0.05) (Fig. 2).

**Figure 3:**
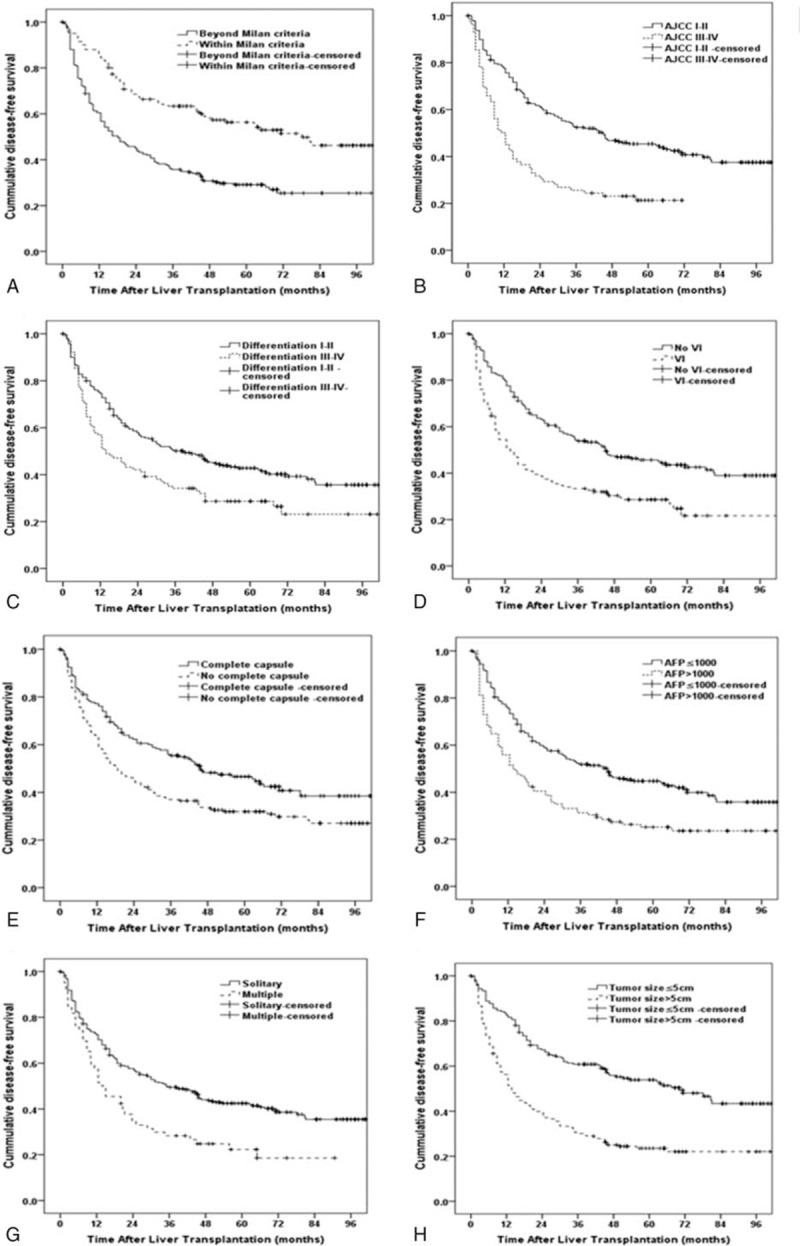
Disease-free survival stratified by (A) Milan criteria (log-rank *P* < 0.001), (B) AJCC stage (log-rank P < 0.001),(C) differentiation (log-rank *P* = 0.002), (D) vascular invasion (log-rank *P* < 0.001), (E) capsule (log-rank *P* = 0.002), (F) AFP (log-rank *P* = 0.001), (G) tumor number (log-rank *P* < 0.001), (H) tumor size (log-rank *P* < 0.001).

As shown in Tables 2 and 5, actuarial DFS and CDFS3 rates were stratified by different clinicopathologic variables such as age, sex, viral hepatitis, cirrhosis, Milan criteria, AJCC stage, differentiation, microvascular invasion, capsule, AFP, tumor number, and tumor size. The Kaplan–Meier analysis suggested that AFP, tumor number, tumor size, capsule, microvascular invasion, differentiation, AJCC, and Milan criteria were associated with decreased actuarial DFS (all *P* < 0.05; Table 2, Fig. 3).

**Table 5:**
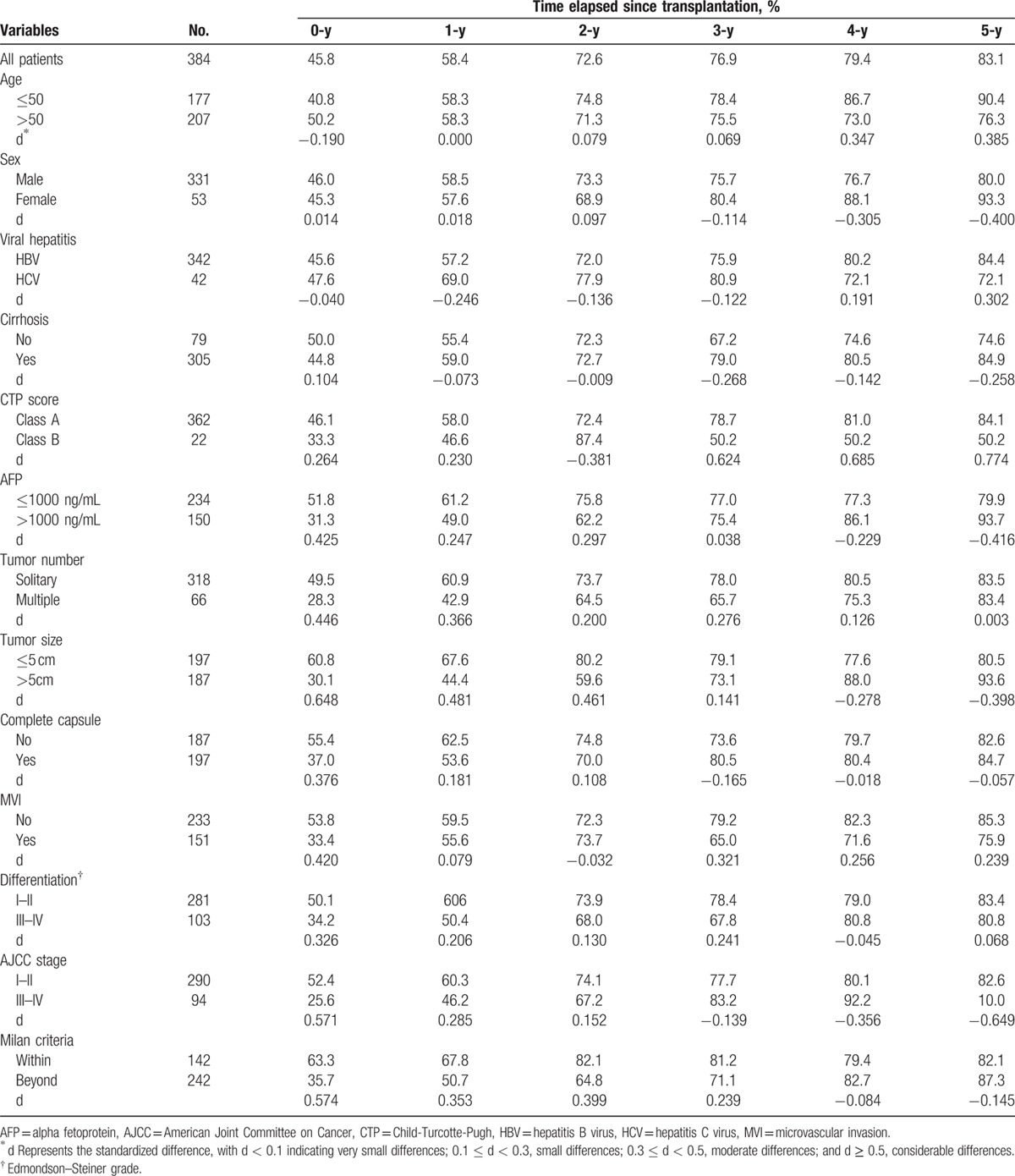
Three-year conditional disease-free survival rates in relationship to patients’ characteristics.

**Figure 4:**
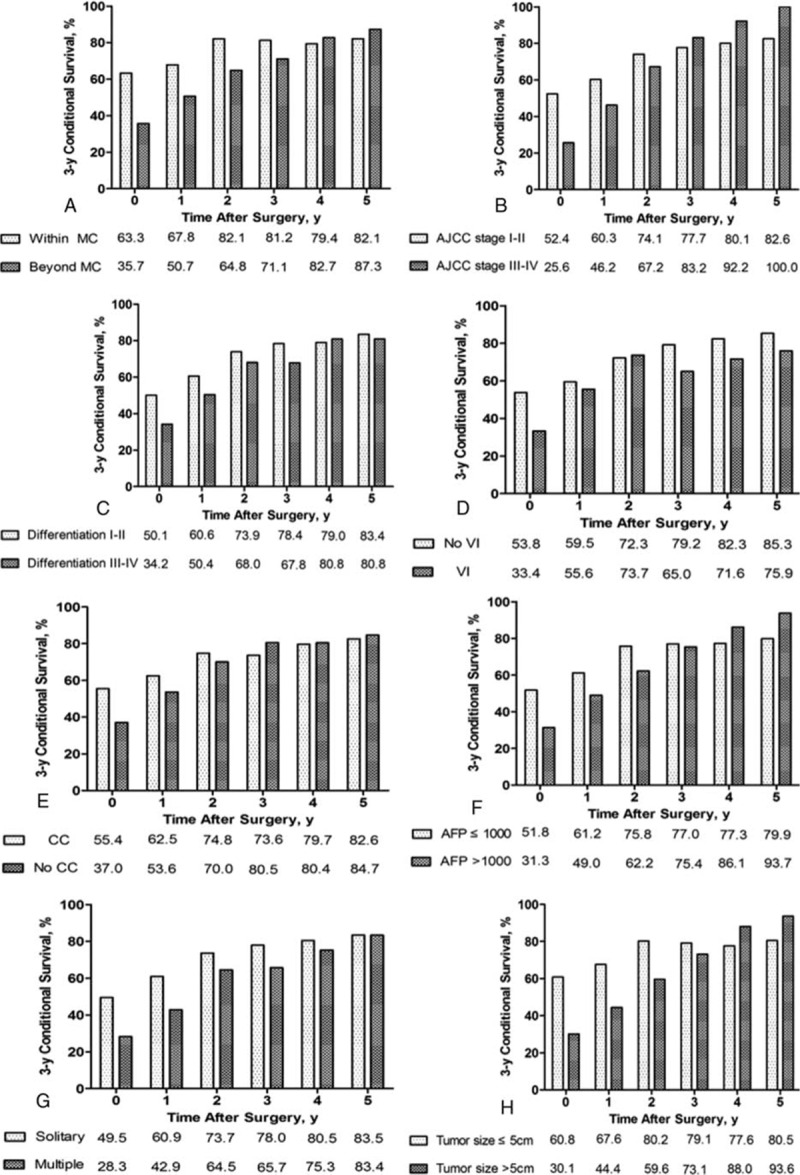
Conditional disease-free survival estimates stratified by (A) Milan criteria, (B) AJCC stage, (C) differentiation, (D) vascular invasion, (E) capsule, (F) AFP, (G) tumor number, (H) tumor size.

The calculated CDFS3 exceeded the actuarial DFS in all corresponding subgroups. Furthermore, this difference was more obvious for those patients who were initially predicted to have poor prognosis. For example, patients with microvascular invasion had a CDFS3 of 75.9% at 5 years compared with an actuarial DFS of 21.7 % at 6 years (Δ54.2%). Similarly, patients with larger tumors (>5cm) had a CDFS3 of 93.6% at 5 years compared with an actuarial DFS of 22.0% at 6 years (Δ71.6%). Conversely, patients characterized by lower risk tumor characteristics had smaller differences between DFS and CDFS estimates. For instance, patients without microvascular invasion had an actuarial 8-year DFS of 39% compared with a 5-year CDFS3 of 85.3% (Δ46.3 %). By the same token, patients with smaller tumor size (≤5 cm) had a 5-year CDFS3 of 80.5 % compared with an actuarial DFS of 43.4 % at 8 years (Δ37.1 %) (Fig. 4).

CDFS increased over time after surgery (Table 5). Furthermore, the differences of 5-year CDFS3 over time were more substantial for patients with the worse initial prognostic parameters. For example, CDFS3 increased over time in patients with larger tumors (>5 cm) (30.1%–93.6%; Δ63.5%). Smaller changes over time in 5-year CDFS3 were seen in patients with the better initial prognostic parameters. For instance, CDFS3 increased over time in patients with smaller tumors (≤5 cm) (60.8%–80.5%; Δ19.7%).

## 4 Discussion

Liver transplantation has evolved rapidly since the first successful LT performed in 1967 by Thomas Starzl.^[17]^ Traditional survival data provide information of the overall survival and disease-free survival for patients. However, during the follow-up period, the postoperative survivors are concerned about the probability of surviving the next some years given having already survived a period of time after LT. The conditional survival rate can help surgeons answer this important question to patients. Conditional survival which quantifies a patient's changing risk over time is a major concern for patients, clinicians, and researchers. A number of previous studies assessed conditional survival and CDFS among patients with different cancers including ovarian cancer,^[18]^ gastric cancer,^[5]^ appendiceal neoplasms,^[19]^ hepatocellular carcinoma,^[14]^ intrahepatic cholangiocarcinoma,^[12]^ and metastatic renal-cell carcinoma^[8]^; no similar data currently exist concerning the prognosis of patients with HCC following LT. To the best of our knowledge, it is the first study to assess CDFS among patients with HCC after LT. We have demonstrated that CDFS can provide more accurate prognostic information for patients who have survived 1 year or longer. Specifically, we noted that CDFS estimates exceeded actuarial DFS estimates among patients who had survived for a period since discharge.

In the present study, we also found that CDFS increased over time after LT. However, several prior studies have shown that the conditional survival rate of different variable after liver resection or radiofrequency ablation (RFA) all increased in the first 2 years, and then decreased in the coming years.^[14,20]^ This could be due to the fact that early recurrence after hepatic resection or RFA might primarily represent metastasis from the primary tumor, whereas late recurrence after hepatic resection or RFA is most likely due to a multicentric occurrence.^[21]^ Since LT removes both HCC and chronic hepatopathy, CDFS after LT increases over time. The difference between CDFS and DFS after LT could also be due to the fact that HCC recurrence mostly occurs within 2 years.^[9]^ Thus, survivors without recurrence until 3 year after transplantation would rarely experience HCC recurrence thereafter. This result supports a more aggressive use of LT for HCC patients.

A large number of previous studies have analyzed clinicopathologic factors associated with prognosis among HCC patients undergoing LT.^[22–24]^ Various factors such as tumor size, tumor number, Milan criteria, AJCC stage, Edmondson–Steiner Grade, AFP, or microvascular invasion have been confirmed to be associated with long-term prognosis.^[25–28]^ In this study, we identified several factors associated with poor DFS by the Kaplan–Meier analysis. Patients with these adverse prognostic features need closer surveillance and/or more aggressive adjuvant treatment. What interests us more is that HCC patients with these adverse prognostic factors were associated with worse actuarial DFS, but the improvements in CDFS of these patients were also greater than the patients with favorable prognostic factors. For example, over a 5-year period, patients with larger tumors (>5 cm) demonstrated a 63.5% increase in CDFS3 estimates versus only a 19.7% increase for patients with smaller tumors. Similar results were seen for the Milan criteria, microvascular invasion, and AJCC stage and so on. Spolverato et al^[12]^ put forward an explanation for this phenomenon: many patients with these adverse prognostic factors die within the first few years after surgery, which results in pessimistic actuarial overall survival. However, some high-risk patients pass through the crisis of this illness, and the adverse prognostic factors at the time of surgery become increasingly less relevant as that specific patient accrues years lived.^[12]^ Therefore, patients remaining alive for the first few years have the similar survival estimates as patients with more favorable tumor features. We also found that the AJCC stage and CTP score had the higher standardized differences of the 5-year CDFS3 (d value greater than 0.5). A better understanding of the role played by these variables on survival at different time points after surgery can help doctors decide the time and length of therapy, adjuvant treatments, and postoperation follow-up.^[14]^ For this reason, CDFS estimates should be used in these patients particularly with high risk in clinical practice.

The current study has several limitations that should be considered. As with all retrospective studies, there may have been selection bias regarding the diagnosis, treatment, and follow-up of patients in the cohort. More information, such as demographic and lifestyle characteristics (race, smoking status, alcohol use, family history),^[18,29–32]^ adjuvant therapy, and their potential impacts on actuarial DFS and CDFS estimates, were also not analyzed. However, the aim of the current study was to assess the differences between actuarial DFS and CDFS estimates. The lack of this information should not have impacted our ability to achieve this objective.

## 5 Conclusions

Survival estimates following LT of HCC patients change according to survival time accrued since surgery. CDFS estimates improved dramatically over time especially to patients with poorer prognostic features at the time of LT. CDFS estimates may provide the changing probability of survival which permits physicians to assess the individual risk of HCC patients undergoing LT and facilitates risk communications between physicians and patients.
